# Myosteatosis is associated with adiposity, metabolic derangements and mortality in patients with chronic kidney disease

**DOI:** 10.1038/s41430-024-01551-4

**Published:** 2025-01-02

**Authors:** Alice Sabatino, Antonio C. Cordeiro, Carla M. Prado, Bengt Lindholm, Peter Stenvinkel, Carla Maria Avesani

**Affiliations:** 1 Division of Nephrology, Department of Medicine and Surgery, University of Parma, Parma, Italy; 2 Baxter Novum, Division of Renal Medicine, Department of Clinical Science Intervention and Technology, Karolinska Institute, Stockholm, Sweden; 3 HCor, Sao Paulo, Brazil; 4 Department of Agricultural, Food and Nutritional Science, University of Alberta, Edmonton, AB Canada

**Keywords:** Nutrition, Kidney diseases, Metabolic syndrome, Computed tomography

## Abstract

**Background/Objectives:**

Myosteatosis has been associated with sarcopenia, and increased mortality risk in patients on hemodialysis. We aimed to explore the associations between myosteatosis, as assessed by computed tomography (CT), with demographic parameters, body composition metrics, muscle strength, metabolic parameters and mortality in patients with chronic kidney disease (CKD).

**Subjects/Methods:**

We enrolled 216 patients (age 60.3 ± 10.6 years, 63% men) with CKD stages 3–5. Abdominal CT scans at the third lumbar vertebra (L3) were used to assess body composition. Abdominal obesity was determined by abdominal adipose tissue (AT), sarcopenia by low skeletal muscle area (SMA) and low handgrip strength. Myosteatosis was evaluated by two parameters using CT scans at L3: mean muscle attenuation and percentage of intermuscular adipose tissue (%IMAT) within SMA. We evaluated the correlation between parameters of myosteatosis with demographic, clinical and metabolic variables. To determine independent predictors of myosteatosis, a multiple linear regression model was fitted. Mortality risk was evaluated with Cox-regression analysis.

**Results:**

Both parameters of myosteatosis were independently associated with age, metabolic syndrome, abdominal AT and SMA in the multiple linear regression analysis (adjusted R^2^ for multiple linear regression: muscle attenuation model 0.535, *P* < 0.001; %IMAT model 0.462, *P* < 0.001). Moreover, higher %IMAT and lower attenuation were associated with a higher mortality risk.

**Conclusion:**

In patients with CKD, increased myosteatosis, as assessed by abdominal CT, was associated with old age, adiposity, metabolic dysfunction, and higher mortality risk.

## Introduction

Myosteatosis is defined as an ectopic deposition of adipose tissue in the skeletal muscle between muscle fibers (intermuscular adipose tissue, IMAT) or within muscle fibers and myocytes (intramuscular adipose tissue) [1]. Myosteatosis has a direct effect on muscle quality by diminishing muscle contractile capacity and reducing muscle function and strength, regardless of muscle quantity [2, 3]. The quantification of myosteatosis is done through muscle biopsy or surrogate markers using imaging techniques including magnetic resonance spectroscopy (MRS), computed tomography (CT) and ultrasound (US) [4]. In addition to its effects on muscle health, evidence suggests a link between myosteatosis and metabolic disturbances, such as insulin resistance, low-grade inflammation, and cardiovascular disease [2, 3, 5–7].

When examining myosteatosis using CT imaging, information can be obtained from trunk images. These images can be opportunistically used to evaluate myosteatosis from the slice at the third lumbar vertebra (L3) [8]. IMAT evaluation in a cross-sectional CT image can be challenging as the image corresponds to a very small compartment that is prone to assessment errors because it is highly dependable on the field of view. In contrast, areas of the muscle with low attenuation values are thought to reflect and mark intramuscular fat infiltration. An alternative assessment method involves calculating the average attenuation of the targeted skeletal muscle.

In the context of chronic kidney disease (CKD), research on myosteatosis is still in its nascent stages. Current evidence shows that patients on hemodialysis exhibit greater myosteatosis and reduced muscle function compared to age- and sex-matched healthy controls [9]. In patients not on dialysis, Kim et al. [10], using CT scans of L3, showed an association between myosteatosis and aortic calcium score, CKD progression and mortality in 149 older patients with CKD. Adverse health events related to myosteatosis in patients with CKD not on dialysis were also shown by Wilkinson et al. [11]. Furthermore, myosteatosis (assessed by MRS) was associated with mitochondrial dysfunction in CKD, indicating a pathophysiological pathway connecting myosteatosis with muscle health [12]. The determinants of myosteatosis and its association with survival rates in patients with CKD not on dialysis have not been carefully explored. Therefore, our aim was to investigate how myosteatosis is associated with other markers of body composition, muscle strength, markers of cardiovascular risk and markers of metabolic dysfunction, in a group of patients with CKD stages 3 to 5. As a secondary outcome, we investigated the association between markers of myosteatosis and survival.

## Materials and methods

### Study design and eligibility criteria

This was an observational longitudinal study conducted as part of the larger *Malnutrition, Inflammation and Vascular Calcification cohort –* MIVC (Dante Pazzanese Institute of Cardiology, São Paulo, Brazil) that aimed to study the association between cardiovascular disease (CVD) and uremic and non-uremic risk factors for CVD. The MIVC included 300 consecutive patients with CKD undergoing regular follow-up in the outpatient clinic of the Hypertension and Nephrology Division at Dante Pazzanese Institute of Cardiology from March 2010 to March 2013. Detailed information on this cohort study can be found elsewhere [13]. According to the eligibility criteria, the main study comprised patients >18 and <80 years, and with a glomerular filtration rate (GFR) (assessed by creatinine clearance with 24-h urine collection) <60 ml/min/1.73 m^2^. The study was conducted according to the Helsinki declaration. Informed consent was signed before their inclusion in the study. The Research Ethical Committee at Dante Pazzanese Institute of Cardiology approved the study (Protocol n° 3846). For this study, we selected 216 patients who had available CT images and complete information on body composition.

### Collected variables

Demographic, clinical, and nutritional data were collected from the original database. Demographic data consisted of age and sex. Clinical data consisted of CKD stage, creatinine clearance, coronary artery calcium (CAC)-score, arterial blood pressure, presence of comorbidities, Charlson comorbidity index (CCI) [14] and biochemical variables. Nutritional parameters of interest were anthropometric parameters (body weight, height), phase angle assessed by bioelectrical impedance analysis (BIA), body composition assessed by CT (skeletal muscle area [SMA], percentage of IMAT [%IMAT], mean muscle attenuation and abdominal adipose tissue [AT]), handgrip strength (HGS), and, additionally, the epicardial adipose tissue evaluated by CT. For detailed description of the methodology for all assessed variables, please see the Supplementary methods.

#### Myosteatosis evaluation

CT images from thoracoabdominal area were used for the assessment of skeletal muscle area (SMA), abdominal adipose tissue, visceral adipose tissue (VAT) and IMAT at L3 with the use of the Slice-O-Matic software version 5.0 (Tomovision, Montreal, Canada). The attenuation value between −29 to +150 HU was used to calculate SMA and the attenuation value between −190 to −30 HU was used to calculate abdominal adipose tissue, VAT and IMAT [15]. Muscle attenuation, a marker of muscle density, was derived by averaging skeletal muscle Hounsfield units (HU), with higher attenuation values indicating lower fat content inside the muscle. Additionally, the percentage of IMAT (%IMAT) was calculated as: IMAT (cm^2^)/ [IMAT (cm^2^) + SMA (cm^2^)] × 100. One skilled dietitian was trained to read all CT images for the assessment of the above mentioned variables. Both IMAT and the average skeletal muscle attenuation were used as markers of myosteatosis.

Considering that there is no cut-off for IMAT derived in Brazilian or European cohorts, only for muscle attenuation [16], we chose to use tertiles of myosteatosis data, separated by sex, for consistency in the statistical analysis for both markers of myosteatosis.

#### Abdominal obesity diagnosis

Patients were considered obese when excess abdominal adipose tissue was present. In the absence of reference values for the Brazilian individuals, we considered reference values that were able to predict mortality in a study of patients undergoing hemodialysis, where the thresholds were: >322.5 cm^2^ for females and >407.8 cm^2^ for males [17].

#### Sarcopenia diagnosis

Low muscle strength was diagnosed when HGS values were <36.6 Kg for males and <20.7 Kg for females, using reference values derived from Brazilian healthy subjects [18]. In the absence of reference values of low muscle mass for Brazilian individuals, patients were diagnosed with low muscle mass when SMA was <125.5 cm^2^ for males and <99.5 cm^2^ for females, using reference values derived from healthy subjects that predicted mortality in patients on hemodialysis [19]. Although other cut-offs derived from healthy subjects are available in the literature [16, 20, 21], we chose these specific cut-offs because they were derived from an Italian cohort, which is phenotypically more similar to our cohort of Brazilian patients from São Paulo. Patients who presented both low HGS and low SMA were considered sarcopenic according to the European consensus definition of sarcopenia [22]. Patients with sarcopenia who also had excess abdominal adipose tissue by CT, were diagnosed as having sarcopenic obesity, which according to approach recently used by our group in a cohort study of patients on hemodialysis, was found to be associated with increased mortality [17].

#### Metabolic syndrome

Metabolic syndrome was defined according to the NCEP ATP III [23], where patients with at least three of the following five criteria were diagnosed with metabolic syndrome: Waist circumference >102 cm for men and >88 cm for women; blood pressure >130/85 mmHg or treated with antihypertensive medication, fasting triglycerides >150 mg/dL, fasting high density lipoprotein (HDL) < 40 mg/dL in men and <50 mg/dL in women and fasting blood sugar >100 mg/dL or using hypoglycemic medication.

### Statistical analysis

Data are expressed as mean and standard deviation (SD) for continuous variables with normal distribution, or median and interquartile range for non-normally distributed data, and as frequencies for categorical variables. Normality was assessed by the Kolmogorov-Smirnov test. Pearson or Spearman coefficients were used to assess the correlation between parameters of myosteatosis with other clinical variables. Multiple linear regression models were fitted based on Pearson’s and Spearman’s correlations results. Non-normally distributed variables were log-transformed to fit the linear regression models.

Patients were divided based on tertiles of %IMAT and muscle attenuation distribution by sex. Between-group differences were analyzed using one-way ANOVA or Kruskal-Wallis one-way ANOVA for continuous variables with Bonferroni corrected post-hoc comparisons, or the Chi-square test for categorical variables and frequencies, as appropriate.

Univariate and multivariable Cox regression analysis were used to assess risk of all-cause mortality associated to %IMAT and muscle attenuation. The multivariable model included as covariates parameters that correlated with myosteatosis at *P* < 0.1 (Supplementary Table 1) and that were also associated with mortality risk at univariate analysis (Supplementary Table 2) following adjustments for age, sex, CAC-score, visceral adipose tissue area, CCI and phase angle.

All analyses were performed using IBM Statistical Package for Social Sciences version 28.0 (IBM SPSS Statistics Inc. Chicago IL. USA). Statistical significance was set at *p* < 0.05 (two-sided).

## Results

**Fig. 1:**
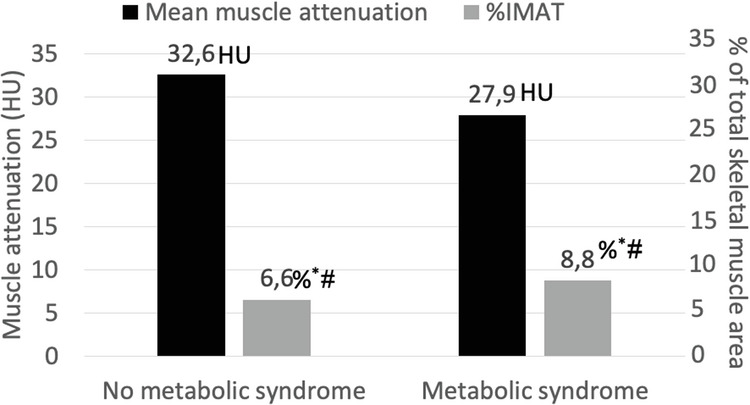
Differences in muscle attenuation (expressed as Hounsfield units, HU) and percentage of intermuscular adipose tissue among 216 non-dialyzed CKD patients with and without metabolic syndrome. %IMAT, percentage of intermuscular adipose tissue. *P < 0.001. ^#^Adjusted by sex and age: *P* < 0.001.

We enrolled 216 outpatients with CKD (Table 1). Patients were 60.3 ± 10.6 years old, with a majority being male (63%). They were evenly distributed across CKD stages 3–5 not on dialysis. The vast majority had hypertension, while half had diabetes. Other common comorbidities in this cohort were peripheral vascular disease and metabolic syndrome, both affecting more than 40% of patients (Table 1). The median CCI was 6 (interquartile range 5–8), which denotes a moderately polymorbid group of patients. Parameters of nutritional status showed that most patients had a BMI in the range of overweight or obesity. Sarcopenia was present in 7.9% of the sample, with the vast majority (76.5%) of those affected being 60 years and older (Table 1). When analyzing the correlation between parameters of myosteatosis as continuous variables with other variables, both were significantly correlated with age, sex, CCI and with variables that are direct or indirect measures of adiposity, and, metabolic syndrome (Supplementary Table 1). Patients with metabolic syndrome had lower muscle attenuation and higher %IMAT (Fig. 1) (*P* < 0.001).

**Table 1:** Demographic and clinical data from the cohort of patients with chronic kidney disease (*n* = 216).

	Patients
Age (years)	60.3 (10.6)
Male (n; %)	136; 63
CKD stage (n; %)	
- Stage 3	74/216 (34.3)
- Stage 4	86/216 (39.8)
- Stage 5	56/216 (25.9)
Creatinine clearance (mL/min/1.73 m^2^)(median, [25^th^–75^th^])	23.7 (14.6–34.4)
Chronic comorbidities (n;%)	
- Diabetes	109; 50.5
- Metabolic syndrome (*n* = 215)	88; 41
Charlson comorbidity index (median, [25^th^–75^th^])	6 (5–8)
Coronary artery calcium score (Agatston) (median, [25^th^–75^th^])	143 (1–517)
Epicardial adipose tissue (cm^2^) (median, [25^th^–75^th^])	1457 (853–2217)
*Laboratory variables*	
Serum creatinine (mg/dL) (median, [25^th^–75^th^])	3.0 (2.3–4.3)
Glycemia (mg/dL) (median, [25^th^–75^th^])	97.5 (85.0–117.7)
Serum albumin (g/dL) (median, [25^th^–75^th^])	3.9 (3.6–4.3)
HbA1C (%)(median, [25^th^–75^th^])	6.4 (5.7–7.6)
Triglycerides (mg/dL) (median, [25^th^–75^th^])	155 (115–222)
25OH-Vitamin D (ng/mL) (median, [25^th^–75^th^])	36.4 (24.4–60.2)
Free testosterone (ng/dL) (median, [25^th^–75^th^])	
- Male	6.4 (4.9–8.0)
- Female	0.3 (0.2–0.6)^b^
C-reactive protein (mg/dL) (median, [25^th^–75^th^])	0.4 (0.1–0.8)
HOMA index (median, [25^th^–75^th^])	2.3 (1.3–4.0)
Leptin (mg/dL) (median, [25^th^–75^th^])	17.9 (6.3–39.5)
*Nutritional status and body composition*	
Body weight (Kg)	
- Male	80.3 (15.9)
- Female	69.7 (15.7)^b^
Body mass index (Kg/m^2^) (median, [25^th^–75^th^])	28.2 (25.0–32.2)
Abdominal obesity by CT (n; %)	13; 6
Waist circumference (cm)	
- Male	98.6 (12.1)
- Female	91.3 (13.4)^b^
Hand-grip strength (Kg)	
- Male	40.1 (9.3)
- Female	27.3 (5.9)^b^
Total abdominal adipose tissue (cm^2^)^a^ (median, [25^th^–75^th^])	
- Male	143.1 (99.7; 193.6)
- Female	204.5 (153.5; 281.2)^b^
Visceral adipose tissue (cm^2^)	
- Male	219.2 (116.8)
- Female	145.8 (86.4)^b^
Phase angle (°)	
- Male	6.3 (1.2)
- Female	5.8 (0.9)^b^
Skeletal muscle area (Kg/m^2^)^a^	
- Male	156.8 (29.8)
- Female	108.4 (17.7)^b^
Muscle attenuation (HU)^a^	
- Male	32.6 (8.5)
- Female	27.2 (7.7)^b^
Intermuscular adipose tissue (cm^2^)^a^ (median, [25^th^–75^th^])	
- Male	7.9 (4.6–13.3)
- Female	10.5 (6.7–16.2)^b^
Intermuscular adipose tissue percentage^a^ (median, [25^th^–75^th^])	
- Male	4.75 (2.89–7.99)
- Female	8.59 (5.79–13.11)^b^
Sarcopenia	
- Low muscle strength (n; %)	59; 27.3
- Low muscle mass (n; %)	41; 19
- Low muscle strength and mass combined (n;%)	17; 7.9
- Sarcopenic obesity (n; %)	2; 0.9

Data provided as mean (SD) except when otherwise specified.

*CKD* Chronic kidney disease, *CT* Computed tomography, *HbA1C* Hemoglobin A1C (glycated hemoglobin), *HGS* Handgrip strength.

^a^Evaluated by computed tomography.

^b^*P* < 0.001 in comparison to males.

**Fig. 2:**
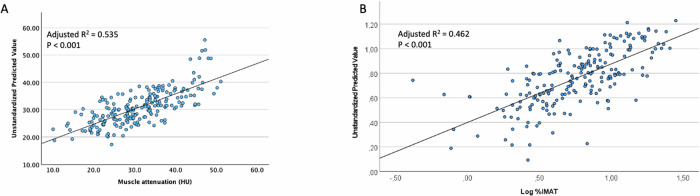
Multiple linear regression analysis of predictors of myosteatosis parameters (*n* = 216). **A** Model for muscle attenuation included age, metabolic syndrome, total abdominal adipose tissue, skeletal muscle area, C-reactive protein, Coronary artery calcium score and epicardial adipose tissue. HU, Hounsfield unit. **B** Model for the percentage of intermuscular adipose tissue included age, Charlson comorbidity index, metabolic syndrome, total abdominal adipose tissue, skeletal muscle area, and phase angle. %IMAT, percentage of intermuscular adipose tissue.

Multiple linear regression models were fitted for both parameters of myosteatosis (Supplementary Table 3). Fig. 2A, B show good prediction of muscle attenuation and %IMAT by the models as shown by the regression lines. Free testosterone was not included in both models due to high collinearity with sex; waist circumference and diabetes were not included as they are part of the diagnostic criteria for metabolic syndrome, and visceral adipose tissue was not included due to high collinearity with total abdominal adipose tissue. Both models effectively predicted the dependent variable, demonstrating large effect sizes (Fig. 2A: adjusted R^2^ = 0.535; *P* < 000.1; Fig. 2B: adjusted R^2^ = 0.462; *P* < 0.001). Greater abdominal adipose tissue, age, presence of metabolic syndrome and SMA remained independent determinants of both myosteatosis markers. Moreover, high epicardial adipose tissue and CRP remained independent predictors of muscle attenuation, and low phase angle and CCI independent predictors of %IMAT.

### Tertiles of myosteatosis

Patients in the 1^st^ tertile of muscle attenuation (representing higher fat infiltration) were older, had lower levels of 25OH-vitamin D, higher comorbidity burden, higher C-reactive protein levels and higher CAC-score (Table 2). This was accompanied by a statistically higher frequency of metabolic syndrome in the 1^st^ and 2^nd^ tertile as compared to 3^rd^ tertile. In terms of body composition, the 1^st^ tertile had higher body fat/adiposity (as indicated by BMI, waist circumference and abdominal adipose tissue) when compared to the 3^rd^ tertile. Furthermore, patients in the 1^st^ tertile had a greater amount of epicardial adipose tissue, and free testosterone was significantly lower in the first tertile in men.

**Table 2:** Demographic, clinical and body composition characteristics of patients with chronic kidney disease by tertile of muscle attenuation (*n* = 216).

Variable	1st tertile muscle attenuation (*n* = 70)	2nd tertile muscle attenuation (*n* = 74)	3rd tertile muscle attenuation (*n* = 72)	*P*
Tertile range (HU)	< 22.6 female	≥22.6 < 30.1female	>30.07 female	NA
	< 28.4 male	≥ 28.4 < 35.6 male	> 35.6 male	
Age (years)	63.8 (9.0)	60.8 (9.2)	56.3 (12.1)^#%^	<0.001
Male (n; %)	44; 63	47; 64	45; 63	0.992
Creatinine clearance (ml/min/1.73 m^2^) (median, [25^th^–75^th^])	24.9 (17.0–32.6)	23.1 (14.3–36.6)	21.7 (14.3–34.7)	0.956
25-OH Vitamin D (pg/mL) (median, [25^th^–75^th^])	36.2 (21.1–60.3)	30.6 (22.4–57.0)	44.3 (28.3–65.5)^%^	0.046
Charlson comorbidity index (median, [25^th^–75^th^])	7.0 (5.7–8.0)	6.5 (5.0–8.0)	6 (5–7.7)^&^	0.037
CAC-score (median, [25^th^–75^th^])	250 (40–1017)	135 (0–404)^£^	29 (0–324)^#*^	0.002
Glycemia (mg/dL)	111.7 (44.0)	109.0 (34.7)	116.1 (74.2)	0.726
HOMA index (median, [25^th^–75^th^])	2.8 (1.5–4.4)	2.3 (1.3–4.0)	2.0 (0.8–3.9)	0.085
HbA1c%	7.0 (1.6)	6.8 (1.6)	7.0 (2.0)	0.586
Free testosterone (ng/dL) (median, [25^th^–75^th^])				
- Male	5.63 (4.20; 7.42)	6.81 (5.05; 7.39)	7.23 (5.45; 8.97)^&^	0.010
- Female	0.32 (0.19; 0.60)	0.35 (0.18; 0.59)	0.37 (0.11; 0.62)	0.664
Serum albumin (mg/dL)	3.9 (0.5)	3.9 (0.5)	3.8 (0.6)	0.530
CRP (g/L) (median, [25^th^–75^th^])	0.5 (0.2–0.8)	0.5 (0.2–1.0)	0.3 (0.1–0.7)^&%^	0.021
Leptin (ng/mL) (median, [25^th^–75^th^])	23.6 (11.0–48.5)	20.6 (11.1–44.7)	7.6 (3.5–24.6)^#^*	<0.001
Tryglycerides (mg/dL) (median, [25^th^–75^th^])	164 (118–223)	160 (120–226)	144 (98–219)	0.313
Metabolic syndrome	35/69 (51%)	38/70 (51%)	15/72 (21%)^#^*	<0.001
Body mass index (kg/m^2^)				
- Female	31.6 (5.5)	31.1 (6.6)	24.7 (4.4)^#^	<0.001
- Male	31.1 (4.5)	29.6 (4.0)	25.5 (4.6)^#^	<0.001
Waist circumference (cm)				
- Female	97.2 (9.6)	96.1 (13.2)	80.8 (10.4)^#^	<0.001
- Male	104.5 (8.5)	100.9 (10.4)	90.3 (12.4)^#^	<0.001
Total abdominal adipose tissue (cm^2^) (median, [25^th^–75^th^])				
- Female	265.9 (206.2; 365.9)	267.6 (187.5; 311.5)^#^	135.3 (99.9; 168.8)^#^	<0.001
- Male	176.6 (141.5; 222.9)	158.6 (116.5; 193.8)^#^	90.1 (60.4; 137.6)^#^	<0.001
Visceral adipose tissue (cm^2^) (median, [25^th^–75^th^])				
- Female	194.0 (125.7; 241.6)	183.6 (95.2; 214.3)^&^	73.5 (22.1; 161.6)^#^	<0.001
- Male	314.8 (241.2; 344.4)	241.4 (178.9; 307.1)^&^	98.3 (44.3; 163.9)^#*^	<0.001
SMA (cm^2^)				
- Female	104.5 (18.3)	113.5 (19.5)	107.1 (14.3)	0.161
- Male	155.4 (29.6)	161.6 (28.6)	153.3 (31.1)	0.387
Phase angle (°)				
- Female	5.9 (1.0)	5.9 (0.8)	5.7 (1.0)	0.828
- Male	6.1 (1.1)	6.2 (1.2)	6.4 (1.3)	0.635
Handgrip Strength (Kg)				
- Female	26.2 (5.4)	28.5 (6.6)	27.0 (5.7)	0.383
- Male	39.4 (3.4)	41.6 (8.1)	39.4 (11.0)	0.420
Epicardial adipose tissue (cm^3^) (median, [25^th^–75^th^])	1853 (1220–2477)	1786 (1125–2426)	764 (537–1532)^#^*	<0.001
Sarcopenia (n; %)	8; 11.4	5; 6.7	4; 5.5	0.390
Deceased (n; %)	25; 36	16; 22	21; 29	0.174

Data provided as mean (SD) except when specified.

*NA* Not applicable, *BIA* Bioelectrical impedance, *CAC* Coronary artery calcium, *CRP* C-reactive protein, *HGS* Handgrip strength, *MAMC* Mid-arm muscle circumference, *SMA* Skeletal muscle area.

^#^*P* < 0.001 in comparison to first tertile; *P < 0.001 in comparison second tertile; ^&^*P* < 0.05 in comparison to first tertile; ^%^*P* < 0.05 in comparison to second tertile; ^£^*P* < 0.01 in comparison to first tertile.

For %IMAT (Table 3), the patients in the 3^rd^ tertile (representing higher intermuscular fat infiltration) were older patients, had higher CAC score and HOMA indices, and a more frequent occurrence of metabolic syndrome. Regarding additional parameters of body composition and anthropometrics, the 3^rd^ tertile of %IMAT showed higher BMI, waist circumference, and abdominal adipose tissue, and, in women, a significantly lower SMA. Additionally, the 3^rd^ tertile had higher epicardial adipose tissue and lower free testosterone in men.

**Table 3:** Demographic, clinical and body composition characteristics of the cohort of patients with chronic kidney disease based on tertile of intermuscular adipose tissue (IMAT) percentage (*n* = 216).

Variable	1^st^ tertile IMAT percent (*n* = 72)	2^nd^ tertile IMAT percent (*n* = 72)	3^rd^ tertile IMAT percent (*n* = 72)	*P*
Tertile range (HU)	<6.6 female	≥6.6 < 11.1female	>11.1 female	NA
	<3.4 male	≥3.4 < 6.8 male	>6.8 male	
Age (years)	55.1 (10.1)	60.8 (10.7)	65.0 (8.7)	<0.001
Male (n; %)	45/72 (62.5)	46/72 (63.9)	45/72 (62.5)	0.980
Creatinine clearance (ml/min/1.73m^2^) (median, [25^th^-75^th^])	20.1 (14.1-32.5)	23.5 (15.8-37.5)	25.9 (16.9–33.9)	0.599
25-OH Vitamin D (pg/mL)	44.5 (27.9–63.7)	34.4 (24.4–53.4)	34.5 (20.0–59.3)	0.075
Charlson comorbidity index	6 (5–8)	5 (5–8)	7 (5–8)	0.146
CAC score	25 (0–250)	184 (5–983)^#^	199 (36–587)^#^	0.002
Glycemia (mg/dL)	114.7 (75.8)	113.6 (39.0)	108.5 (36.8)	0.765
HOMA index	1.75 (0.9–3.1)	2.9 (1.4–5.2)^#^	2.5 (1.3–4.0)	0.006
HbA1c%	7.3 (2.2)	6.8 (1.6)	6.7 (1.4)	0.176
Free testosterone (ng/dL)				
- Male	7.23 (5.30; 8.72)	6.69 (5.01; 8.12)	5.75 (4.20; 7.02)^&^	0.004
- Female	0.32 (0.11; 0.59)	0.36 (0.16; 0.61)	0.33 (0.21; 0.64)	0.426
Serum Albumin (mg/dL)	3.7 (0.6)	4.0 (0.6)^&^	3.9 (0.5)	0.023
CRP (g/L)	0.30 (0.09–0.79)	0.49 (0.15–0.81)	0.48 (0.15–0.83)	0.236
Leptin (ng/mL)	10.2 (3.5–24.2)	19.3 (10.5–42.2)^#^	23.4 (10.9–49.8)^#^	<0.001
Tryglycerides (mg/dL)	139 (110–209)	173 (118–235)	158 (116–211)	0.559
Metabolic syndrome	17/72 (23.6%)	34/72 (47.2%)	37/71 (52.1%)	0.001
BMI (kg/m [2])				
- Female	25.3 (4.2)	30.5 (6.3)^#^	31.5 (6.6)^#^	<0.001
- Male	25.9 (3.6)	29.3 (4.7)^#^	30.9 (5.0)^#^	<0.001
Waist circumference (cm)				
- Female	83.1 (10.2)	94.1 (12.6)^#^	96.8 (13.4)^#^	<0.001
- Male	90.6 (9.3)	100.3 (11.0)^#^	104.8 (11.3)^#^	<0.001
Total abdominal adipose tissue (cm [2])				
- Female	152.2 (109.7; 200.7)	223.7 (181.0; 284.4)^&^	260.2 (200.8; 360.9)^#^	<0.001
- Male	98.7 (72.4; 144.2)	142.7 (114.3; 248.1)^#^	192.8 (141.8; 224.8)^#^	<0.001
Visceral adipose tissue (cm [2])				
- Female	73.5 (22.1; 161.9)	180.5 (106.4; 232.9)^&^	180.1 (84.8; 239.6)^&^	<0.001
- Male	103.5 (70.1; 168.6)	244.1 (159.1; 328.5)^#^	307.9 (243.5; 344.1)^#&^	<0.001
SMA (CT) (cm [2])				
- Female	110.0 (14.4)	114.3 (19.2)	101.2 (17.3)^£^	0.021
- Male	160.7 (26.6)	156.6 (31.7)	153.2 (30.9)	0.498
Phase angle (°)				
- Female	5.9 (1.0)	5.9 (0.8)	5.7 (1.0)	0.980
- Male	6.4 (1.3)	6.3 (1.3)	6.1 (1.0)	0.090
Handgrip strength (Kg)				
- Female	27.0 (6.1)	28.8 (6.5)	26.0 (5.0)	0.243
- Male	40.3 (10.5)	39.8 (8.7)	40.3 (8.6)	0.947
Epicardial adipose tissue (cm [3])	770 (552–1457)	1740 (1184–2348)^#^	1971 (1263–2917)^#^	<0.001
Sarcopenia (n;%)	2; 2.7	4; 5.5	11; 15.2^&^	0.014
Deceased (n; %)	19; 26	18; 25	25; 35	0.378

Data provided as mean (SD) except when specified.

*NA* Not applicable, *CAC* Coronary artery calcium, *CRP* C-reactive protein, *HGS* Handgrip strength, *MAMC* Mid-arm muscle circumference, *SMA* Skeletal muscle area.

^#^*P* < 0.001 in comparison to first tertile; *P < 0.001 in comparison second tertile; ^&^*P* < 0.05 in comparison to first tertile; ^£^*P* < 0.05 in comparison to second tertile.

### Myosteatosis parameters and mortality

**Fig. 3:**
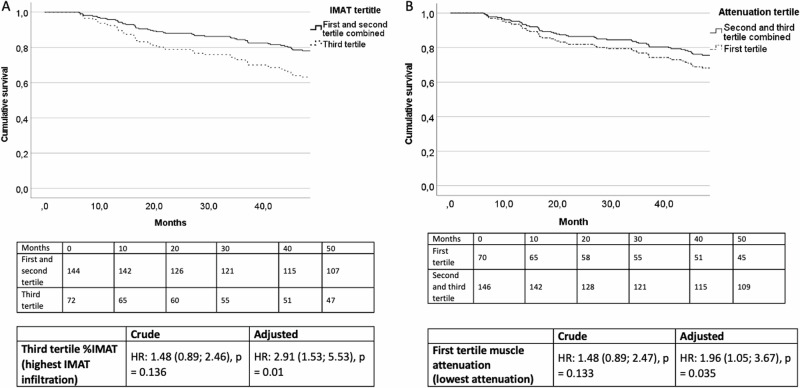
Adjusted probability of survival curve with Cox-regression hazard ratios for mortality (*n* = 216). **A** Adjusted probability of survival for the first tertile of muscle attenuation. **B** Adjusted probability of survival forthe third tertile of percentage of intermuscular adipose tissue, %IMAT.

Patients were followed for median 48 (25^th^–75^th^: 36–48) months. During this period, 62 (29%) patients died. Upon Cox regression analysis, every 10% increase of %IMAT was associated with almost three-fold increased mortality risk while every 10 units increase in muscle attenuation was associated with 42% decrease in mortality risk (Supplementary Table 4). When evaluated categorically (Fig. 3A, B), the highest content of %IMAT and the lowest attenuation tertile were associated with increased mortality risk in comparison to the other two tertiles combined in the adjusted analysis. All analyses were adjusted for age, sex, CAC-score, CCI, visceral adipose tissue and phase angle.

## Discussion

In the present study, we used CT images to assess body composition at the L3 level in a cohort of patients with CKD stages 3–5 to explore factors associated with two CT-derived markers of myosteatosis, %IMAT and average muscle attenuation. This approach allowed us to show that myosteatosis in this cohort of carefully phenotyped patients was associated with several nutritional and metabolic parameters including older age, increased adiposity, and obesity-related metabolic alterations. Furthermore, higher %IMAT and lower attenuation were associated with a higher mortality risk.

Similar findings have been reported in the general population. In a large well-controlled cohort of healthy subjects, Delmonico et al. [24] were the first to demonstrate the increase in myosteatosis with age, a phenomenon that occurred regardless of body weight and muscle mass gain or loss. In the context of CKD, it is probable that factors beyond age contribute to the development of myosteatosis. Evidence shows that patients with advanced CKD on dialysis have increased myosteatosis, as assessed by MRI, compared to age-matched controls [9]. Additionally, Kim et al. [10] identified myosteatosis, assessed by muscle density, as an independent predictor of CKD progression in 149 patients, with a median follow-up of 7.5 years. In our study, we did not find any association between creatinine clearance and myosteatosis. However, we did not evaluate changes in renal function over time, which limits our ability to confirm previous findings. Nevertheless, individuals with CKD often have several conditions already associated with myosteatosis. Available evidence suggests that aging, poor nutritional status, inflammation, oxidative stress, mitochondrial dysfunction, and insulin resistance might act synergistically in the development of myosteatosis [25]. Obesity also seems to be a factor associated with myosteatosis in patients with CKD. In fact, our group has recently shown the association between myosteatosis and other markers of body composition in hemodialysis patients [17]. In the aforementioned work, among four body composition phenotypes (normal, sarcopenia only, obesity only, and sarcopenic obesity), both groups with obesity had the highest prevalence of myosteatosis in comparison to the group with normal body composition [17]. Research involving other patient populations, as well as healthy individuals, further supports a link between obesity and its associated metabolic disturbances and the occurrence of myosteatosis [5, 6, 26]. One possible explanation for this association is that with increased adiposity, adipocytes may exceed their fat-storage capacity, resulting in the accumulation of ectopic fat in lean tissues, including skeletal muscle, liver, and pancreas [3].

Myosteatosis by CT is currently evaluated by assessing IMAT or muscle attenuation. While %IMAT reflects only the intermuscular adipose tissue (i.e., adipose tissue in between muscle fibers and muscle groups), the average muscle attenuation is a measure of muscle density and reflects adipose tissue within skeletal muscle fibers and muscle cells. Lower values of muscle attenuation reflect a greater amount of intramuscular adipose tissue, which will consequently influence muscle density. In fact, a study comparing CT and MR, has shown that, intramyocellular lipid stores rather than extramyocellular lipid stores, better reflected CT-assessed muscle attenuation [27]. Despite limitations in assessing IMAT through a single cross-sectional CT area, the differentiation between adipose tissue outside and inside the fibers may be important because they might have different effects on muscle and metabolic health [3]. A study using MR suggested that intramyocellular lipids rather than extramyocellular lipids influences insulin resistance [28]. However, in studies using CT, both parameters have been shown to be related to inflammatory markers [12, 25], and insulin resistance [29]. In our study, CRP and HOMA index (borderline significance) were higher in the lower tertile of muscle attenuation and, in the case of HOMA index, it was higher in the third tertile of %IMAT. At multivariable linear regression, CRP remained independently associated with muscle attenuation. More in general, CCI was associated with both parameters at univariate analysis, and with %IMAT in multivariable linear regression, while metabolic syndrome remained an independent predictor of both markers of myosteatosis in multivariable linear regression analysis. Similar observations were found in studies involving subjects with type-2 diabetes [5, 6]. Particularly, a recent systematic review showed increased presence of myosteatosis (defined both by IMAT and muscle density) in subjects with diabetes, and its association with insulin resistance [5]. Low muscle density has also been associated with low-grade inflammation [26]. In fact, ectopic adipose tissue in muscles secretes cytokines, leading to localized inflammation [2]. However, it is still unclear whether myosteatosis is solely a marker of metabolic derangements, or if it plays a role in the development of insulin resistance and inflammation.

The association between myosteatosis and health outcomes has also been investigated in relation to increased cardiovascular risk and mortality. Obesity, diabetes, inflammation, and dyslipidemia are known cardiovascular risk factors. Myosteatosis, too, is linked with such risks; studies show it is associated with increased cardiovascular mortality in older men [30], and with higher CAC-scores [31]. Additionally, myosteatosis predicts cardiovascular events and mortality in hemodialysis patients [32], unlike low skeletal muscle mass [33]. In the present study, CAC-score (a well-established marker of increased cardiovascular risk) was associated with both investigated parameters of myosteatosis. At adjusted analysis, all-cause mortality risk was also increased for patients with higher %IMAT and lower muscle attenuation, both when parameters were considered as continuous and categorical variables, but only %IMAT was associated with cardiovascular mortality. Considering the low number of cardiovascular events in non-dialysis CKD cohorts, a larger cohort may be needed to confirm the association between cardiovascular mortality and myosteatosis parameters.

Our study has limitations. Firstly, in order to determine the severity of myosteatosis, we divided the cohort into tertiles, which was arbitrary but allowed the identification of metabolic differences between the worst tertiles and the best tertiles. Secondly, because this is a secondary analysis of a larger study and includes a reduced number of patients from the original cohort, coupled with the intrinsic characteristics of patients with CKD, there might be insufficient statistical power to discern certain differences in hard outcomes. Nevertheless, we were able to show important associations between myosteatosis parameters, metabolic derangements and all-cause mortality in a group of patients with non-dialysis CKD. Finally, our study used CT to evaluate myosteatosis, which, despite being considered a gold standard, is not without limitations. CT-assessed muscle density could be affected by fluid retention. Fluid retention is usually a complication in advanced stages of CKD, however, patients with non-dialysis CKD seldom have fluid overload in an extent that could influence the abdominal muscle density.

In conclusion, in patients with CKD the extent of CT-assessed abdominal myosteatosis was associated with higher age, abdominal adiposity, and markers of metabolic dysfunction. Moreover, in the adjusted analysis, higher %IMAT and lower attenuation were associated with a higher mortality risk.

## Supplementary information

Supplementary material clean

## Data Availability

The data used for this research can be available upon reasonable request.
