# Supplementary material for: Myosteatosis is associated with adiposity, metabolic derangements and mortality in patients with chronic kidney disease
Source: Eur J Clin Nutr. 2025 Jan 2;79(5):475–83. doi: 10.1038/s41430-024-01551-4 (PMC12069101; doi:10.1038/s41430-024-01551-4)
Supplement: Supplementary file 1 — Supplementary material clean [file 41430_2024_1551_MOESM1_ESM.docx]

**Myosteatosis is associated with adiposity, metabolic derangements and mortality in patients with CKD**

Alice Sabatino^1, 2^, Antonio C Cordeiro^3^, Carla M. Prado^4^, Bengt Lindholm^2^, Peter Stenvinkel^2^, Carla Maria Avesani^2^

^1^Division of Nephrology, Department of Medicine and Surgery, University of Parma, Parma, Italy.

^2^Division of Renal Medicine and Baxter Novum, Department of Clinical Science, Technology and Intervention, Karolinska Institute, Stockholm, Sweden

^3^ HCor, Sao Paulo, Brazil

^4^Department of Agricultural, Food and Nutritional Science, University of Alberta, Canada

**Supplementary materials**

**Supplementary Methods**

*Anthropometry, bioimpedance analysis, handgrip strength and subjective global assessment*

All included participants had their body weight, height, triceps skinfold thickness and waist and arm circumferences assessed. Body mass index (BMI) was calculated as body weight in kg divided by height in meter squared and classified according to WHO criteria. Waist circumference was assessed at the midpoint between the lowest lateral border of the ribs and the uppermost lateral iliac crest. Phase angle was quantified using a tetrapolar single frequency bioimpedance analysis (BIA) device (Biodynamics^®^ BIA 450 Bioimpedance Analyzer, Seattle, USA). The same experienced dietitian performed all the anthropometric measurements, and BIA.

Handgrip strength (HGS) was assessed by a handgrip dynamometer (Baseline^®^, NexGen Ergonomics, Inc, Quebec, Canada) in the dominant hand and the highest value of three measurements was considered for the study.

*Computed tomography*

For epicardial adipose tissue, a CT attenuation between –200 and –20 HU was used to isolate the area[1]. First, the reader manually traced the pericardium. Then, any adipose tissue located within the pericardial sac was defined as epicardial adipose tissue. This procedure was performed by an experienced radiologist. CAC-score was determined by a single radiologist using VITREA 2 software, based on the Agatston method[2]. Coronary calcification was identified as a lesion with an area >1 mm^2^ and a peak intensity >130 HU; the score was obtained by multiplying the area of each calcified lesion by a weighting factor corresponding to the peak pixel intensity for the lesion. An Agatston score of ≥10 was chosen to define positive CAC-score due to the high interscan variability within the 0 to 10 range. For the present analysis, total CAC-score was used (i.e. the sum of the scores for each coronary artery). Patients with coronary artery stents (n = 23) did not undergo this examination.

*Laboratory measurements*

Patients had fasting blood samples drawn in the morning and stored at -70 °C. Plasma 25-hydroxy-vitamin D (25-OH vitamin D) was assessed by high-performance liquid chromatography, serum leptin by a commercial enzyme-linked immunosorbent assay kit (LDN, Nordhorn, Germany), and high-sensitivity C-reactive protein (CRP) by immunoturbidimetry. Total cholesterol, high-density lipoprotein cholesterol, triglycerides, creatinine, glycemia, HbA1c and albumin were analysed using validated methods at the Dept. of Laboratory Medicine at Dante Pazzanese Institute of Cardiology. Total serum testosterone and sex  hormone-binding globulin (SHBG) were assessed by chemiluminescence; while free testosterone was calculated from total testosterone, SHBG and albumin according to a previous validated equation[3].

**Methods references**

1. Cordeiro A, Amparo F, Oliveira M*, et al.* Epicardial fat accumulation, cardiometabolic profile and cardiovascular events in patients with stages 3-5 chronic kidney disease. J Intern Med 2015;278(1):77-87

2. Agatston A, Janowitz W, Hildner F*, et al.* Quantification of coronary artery calcium using ultrafast computed tomography. J Am Coll Cardiol 1990;15(4):827-832

3. Vermeulen A, Verdonck L, Kaufman J. A critical evaluation of simple methods for the estimation of free testosterone in serum. J Clin Endocrinol Metab 1999;84(10):3666-3672

**Supplementary Table 1**. Correlations between clinical/body composition parameters and myosteatosis (muscle attenuation/intermuscular adipose tissue) (n=216).

|  | **Muscle attenuation (HU)** | | **% Intermuscular adipose tissue** | |
| --- | --- | --- | --- | --- |
| **Variable** | **R** | **P-value** | **R** | **P-value** |
| Age | -0.38 | < 0.001 | 0.38 | < 0.001 |
| Sex male | 0.31 | < 0.001 | -0.35 | < 0.001 |
| Charlson comorbidity index | -0.21 | 0.002 | 0.16 | 0.020 |
| Creatinine clearance | -0.02 | 0.785 | -0.02 | 0.77 |
|  |  |  |  |  |
| C-reactive protein | -0.12 | 0.080 | 0.03 | 0.628 |
| Serum albumin | -0.09 | 0.177 | 0.04 | 0.554 |
| HOMA index | -0.14 | 0.039 | 0.10 | 0.147 |
| Glycemia | -0.05 | 0.488 | 0.01 | 0.853 |
| HbA1c | -0.03 | 0.627 | -0.04 | 0.599 |
| Serum triglycerides | -0.05 | 0.433 | 0.01 | 0.837 |
| Leptin | -0.43 | < 0.001 | 0.44 | <0.001 |
| 25OH-Vitamin D | 0.14 | 0.045 | -0.14 | 0.039 |
| Free testosterone | 0.38 | < 0.001 | -0.44 | < 0.001 |
| Waist circumference | -0.41 | < 0.001 | 0.30 | < 0.001 |
| BMI | -0.477 | < 0.001 | 0.378 | < 0.001 |
| Total abdominal adipose tissue | 0.57 | < 0.001 | -0.62 | < 0.001 |
| Visceral adipose tissue | 0.34 | < 0.001 | -0.48 | < 0.001 |
| Skeletal muscle area | -0.39 | < 0.001 | 0.21 | 0.002 |
| Handgrip strength | 0.206 | 0.002 | -0.296 | < 0.001 |
| Phase angle (°) | 0.12 | 0.076 | -0.22 | 0.001 |
| Epicardial adipose tissue (cm^3^) | -0.41 | < 0.001 | 0.36 | < 0.001 |
| CAC-score | -0.21 | 0.002 | 0.16 | 0.021 |
| Metabolic syndrome | -0.267 | < 0.001 | 0.237 | < 0.001 |

BMI, Body mass index; CAC-score: Coronary artery calcium; HGS, Handgrip strength; MAMC, Mid-arm muscle circumference.

**Supplementary Table 2.** Hazard ratios showing univariate associations between clinical parameters and body composition variables (that correlated with at least one of the myosteatosis parameters at P < 0.1) with all cause-mortality (n = 216).

|  | HR (95% CI), P |
| --- | --- |
| Age | 1.01 (0.98; 1.04), P = 0.401 |
| Sex (male) | 0.73 (0.44; 1.22), P = 0.227 |
| BMI | 0.99 (0.95; 1.04, P = 0.721 |
| Charlson comorbidity index | 1.17 (1.04; 1.33), P = 0.012 |
| C-reactive protein | 1.47 (0.92; 2.34), P = 0.108 |
| HOMA index | 1.04 (0.54; 1.99), P = 0.917 |
| Leptin | 0.99 (0.99; 1.01), P = 0.841 |
| Serum Triglycerides | 1.00 (0.99; 1.00), P = 0.228 |
| 25OH-Vitamin D | 0.74 (0.26; 1.99), P = 0.551 |
| Free testosterone | 0.94 (0.88; 1.02), P = 0.132 |
| Waist circumference | 0.99 (0.98; 1.02), P = 0.789 |
| Total abdominal adipose tissue | 1.01 (0.99; 1.00), P = 0.789 |
| Visceral adipose tissue | 0.997 (0.995; 0.999), P = 0.017 |
| Skeletal muscle area | 0.99 (0.98; 1.00), P = 0.066 |
| Handgrip strength | 0.95 (0.92; 0.98), P < 0.001 |
| Phase angle | 0.70 (0.55; 0.90), P = 0.006 |
| Epicardial adipose tissue | 0.61 (0.26; 1.42), P = 0.249 |
| CAC-score | 1.34 (1.08; 1.66), P = 0.008 |
| Metabolic syndrome | 1.15 (0.69; 1.92), P = 0.600 |

BMI, Body mass index; CAC-score: Coronary artery calcium score; Univariate Cox-regression analysis for individual variables.

**Supplementary Table 3.** Multiple linear regression analysis for variables predicting skeletal muscle attenuation and percentage of intermuscular adipose tissue in the cohort of patients with chronic kidney disease (n=216).

|  | **Muscle Attenuation** | | | | | | **%IMAT** | | | | | |
| --- | --- | --- | --- | --- | --- | --- | --- | --- | --- | --- | --- | --- |
|  | **Model 1** | | | **Model 2** | | | **Model 1** | | | **Model 2** | | |
| **Variable** | **B** | **SE B** | **P-value** | **B** | **SE B** | **P-value** | **B** | **SE B** | **P-value** | **B** | **SE B** | **P-value** |
| Age (years) | -0.210 | 0.061 | < 0.001 | -0.181 | 0.046 | < 0.001 | 0.009 | 0.002 | <0.001 | 0.011 | 0.002 | < 0.001 |
| Male sex | -0.268 | 1. 556 | 0.864 | - | - | - | 0.110 | 0.064 | 0.087 | - | - | - |
| CRP(log) | -1.311 | 0.774 | 0.092 | -1.653 | 0.728 | 0.024 | - | - | - | - | - | - |
| HOMA index (log) | 2.029 | 1.384 | 0.144 | - | - | - | - | - | - | - | - | - |
| 25-OH Vitamin D (log) | 0.906 | 1.835 | 0.622 | - | - | - | -0.085 | 0.075 | 0.259 | - | - | - |
| Leptin (log) | -1.587 | 1.238 | 0.202 | - | - | - | 0.100 | 0.049 | 0.043 | - | - | - |
| Charlson CI | 0.364 | 0.309 | 0.241 | - | - | - | -0.032 | 0.012 | 0.010 | -0.031 | 0.12 | 0.008 |
| CAC-score (log) | -0.755 | 0.389 | 0.054 | -0.425 | 0.356 | 0.234 | 0.019 | 0.016 | 0.229 | - | - | - |
| Metabolic syndrome | -2.827 | 1.006 | 0.006 | -2.026 | 0.876 | 0.022 | 0.126 | 0.039 | 0.002 | 0.126 | 0.037 | < 0.001 |
| TA Adipose Tissue (log) | -13.260 | 2.210 | < 0.001 | -14.542 | 1.594 | < 0.001 | 0.373 | 0.090 | < 0.001 | 0.458 | 0.060 | < 0.001 |
| Skeletal muscle area | 0.044 | 0.020 | 0.028 | 0.056 | 0.012 | < 0.001 | -0.004 | 0.001 | < 0.001 | -0.003 | 0.001 | < 0.001 |
| Phase angle | 0.614 | 0.441 | 0.165 | - | - | - | -0.046 | 0.018 | 0.010 | -0.039 | 0.017 | 0.023 |
| Epicardial adipose tissue (log) | -3.363 | 1.930 | 0.083 | -3.934 | 1.690 | 0.021 | 0.058 | 0.078 | 0.461 | - | - | - |
| Adjusted R^2^ | 0.540 | | | 0.535 | | | 0.480 | | | 0.462 | | |
| F | 18.25 | | | 35.05 | | | 17.02 | | | 30.06 | | |
| P-value of the model | < 0.001 | | | < 0.001 | | | < 0.001 | | | < 0.001 | | |

Charlson CI: Charlson comorbidity index; CAC-score: Coronary artery calcification score; CRP, C-reactive protein; TA Adipose T: Total abdominal adipose tissue; CRP, C-reactive protein; %IMAT: Percentage of intermuscular adipose tissue; SE: Standard error. Variable that did not have a normale distribution were log transformed to be included in the model.

Muscle attenuation Model 1: Included all variables that were associated with muscle attenuation at univariate analysis.

Muscle attenuation Model 2: Included only the variables that were still statistically significant at model 1, that are age, metabolic syndrome, total abdominal adipose tissue, SMA, and epicardial adipose tissue.

% IMAT Model 1: Included all variables that were associated with %IMAT at univariate analysis.

% IMAT Model 2: Included only the variables that were still statistically significant at model 1, that are included age, Charlson comorbidity index, metabolic syndrome, total abdominal adipose tissue, SMA, and phase angle.

**Supplementary Table 4.** Hazard-ratios for all-cause mortality and cardiovascular mortality considering the parameters of myosteatosis assessed as continuous variables (n=216).

|  | **Unadjusted** | **Adjusted** |
| --- | --- | --- |
| **All-cause mortality** |  |  |
| Muscle attenuation (HU) (every 10 units increase) | HR: 0.85 (0.62;1.18), p = 0.337 | HR: 0.58 (0.36;0.93), P = 0.025 |
| %IMAT (every 10% increase) | HR: 1.87 (1.19; 2.95), p = 0.007 | HR: 2.99 (1.64; 5.46), P < 0.001 |
| **Cardiovascular mortality** |  |  |
| Muscle attenuation (HU) (every 10 units increase) | HR: 0.80 (0.50; 1.25), P = 0.324 | HR: 0.59 (0.30; 1.13), P = 0.111 |
| %IMAT (every 10% increase) | HR: 1.83 (0.95; 3.51), P = 0.069 | HR: 2.57 (1.15; 5.76), P = 0.021 |

Adjusted: age, sex, CAC-score(log), Charlson comorbidity index, visceral adipose tissue and phase angle, IMAT: Intermuscular adipose tissue.
